# Approximate parameter inference in systems biology using gradient matching: a comparative evaluation

**DOI:** 10.1186/s12938-016-0186-x

**Published:** 2016-07-15

**Authors:** Benn Macdonald, Mu Niu, Simon Rogers, Maurizio Filippone, Dirk Husmeier

**Affiliations:** 1 School of Mathematics and Statistics, University of Glasgow, Glasgow, G12 8QW Scotland; 2 School of Computing Science, University of Glasgow, Glasgow, G12 8RZ Scotland; 3 EURECOM, Sophia Antipolis, France

**Keywords:** Parameter inference, Ordinary differential equations, Gradient matching, Gaussian processes, Parallel tempering, Reproducing kernel Hilbert spaces

## Abstract

**Background:**

A challenging problem in current systems biology is that of parameter inference in biological pathways expressed as coupled ordinary differential equations (ODEs). Conventional methods that repeatedly numerically solve the ODEs have large associated computational costs. Aimed at reducing this cost, new concepts using gradient matching have been proposed, which bypass the need for numerical integration. This paper presents a recently established adaptive gradient matching approach, using Gaussian processes (GPs), combined with a parallel tempering scheme, and conducts a comparative evaluation with current state-of-the-art methods used for parameter inference in ODEs. Among these contemporary methods is a technique based on reproducing kernel Hilbert spaces (RKHS). This has previously shown promising results for parameter estimation, but under lax experimental settings. We look at a range of scenarios to test the robustness of this method. We also change the approach of inferring the penalty parameter from AIC to cross validation to improve the stability of the method.

**Methods:**

Methodology for the recently proposed adaptive gradient matching method using GPs, upon which we build our new method, is provided. Details of a competing method using RKHS are also described here.

**Results:**

We conduct a comparative analysis for the methods described in this paper, using two benchmark ODE systems. The analyses are repeated under different experimental settings, to observe the sensitivity of the techniques.

**Conclusions:**

Our study reveals that for known noise variance, our proposed method based on GPs and parallel tempering achieves overall the best performance. When the noise variance is unknown, the RKHS method proves to be more robust.

## Background

A central objective of current systems biology research is explaining the actions among components in biopathways. A standard approach is to view a biopathway as a network of biochemical reactions, which is modelled as a system of ordinary differential equations (ODEs).

This system can typically be expressed as:

1 $$\begin{aligned} \dot{x}_s= \frac{dx_s(t_i)}{dt_i} = f_s(\mathbf x (t_i),\varvec{\theta }_s,t_i), \end{aligned}$$

where $s\in \{1,\dots ,N\}$ denotes one of *N* components (referred to throughout as “species”) in the biopathway, $x_s(t_i)$ denotes the concentration of species $s$ at time $t_i$ and $\mathbf x (t_i)$ is a vector of concentrations of all system components that influence or regulate the concentration of species $s$ at time $t_i$.^1^ If, for example, species $s$ is an mRNA, then $\mathbf x (t_i)$ might contain the concentrations of transcription factors (proteins), from which $s$ is transcribed, that bind to the promoter of the gene. The regulation is described by the regulation function *f*. The type of regulatory interaction depends on the species involved, e.g., *f* may describe mass action kinetics, Michaelis-Menten kinetics, etc. All of these interactions depend on a vector of kinetic parameters, $\varvec{\theta }_s$. For many biopathways, only a small fraction of $\varvec{\theta }_s$ can be measured in practice. Therefore, in order to understand the dynamics of the biopathway, the majority of these kinetic parameters need to be inferred from observed (typically noisy and sparse) time course concentration profiles.

**Table 1:** Examples of the notation used throughout this paper

Notation	Meaning	Example
Bold face uppercase letter or symbol	Matrix	$\mathbf X$
Bold face lowercase letter or symbol	Vector	$\varvec{\theta }$
Vector at time $t_i$	Concentration for all species at time $t_i$	$\mathbf y (t_i)$ or $\mathbf x (t_i)$
Vector of concentrations for species $s$	Concentrations for species $s$ over all timepoints	$\mathbf y _s$ or $\mathbf x _s$
Vector of concentrations	Concentrations over all timepoints for one species	$\mathbf y$ or $\mathbf x$
Lower case letter at time $t_i$ for species $s$	Concentration for species $s$ at timepoint $t_i$	$y_s(t_i)$ or $x_s(t_i)$

Conventional inference methods typically rely on searching the space of $\varvec{\theta }$ values, and at each candidate, numerically solving the ODEs and comparing the output with that observed. After choosing an appropriate noise model, the form of the likelihood is defined, and a measure of similarity between the data signals and the signals described by the current set of ODE parameters can be calculated. This process is repeated, as part of either an iterative optimisation scheme or sampling procedure in order to estimate the parameters. However, the computational costs involved with repeatedly numerically solving the ODEs are usually high.

Several authors have adopted approaches based on gradient matching (e.g. [1, 2]), aiming to reduce this computational complexity. These approaches are based on the following two-step procedure. At the first step, interpolation is used to smooth the time series data, in order to avoid modelling noisy observations; in a second step, the kinetic parameters $\varvec{\theta }$ of the ODEs are either optimised or sampled, whilst minimising some metric measuring the difference between the slopes of the tangents to the interpolants, and the $\varvec{\theta }$-dependent time derivative from the ODEs. In this fashion, the ODEs never have to be numerically integrated, and the problem of inferring the typically unknown initial conditions of the system is removed, as it is not required for matching gradients. A downside to this two-step scheme is that the results of parameter inference are critically dependent on the quality of the initial interpolant. Alternatively, as first suggested in [3], we can allow the ODEs to regularise the interpolant. Dondelinger et al. [4] applied this to the nonparametric Bayesian approach in [1], which uses Gaussian processes (GPs), and demonstrated that it significantly improves the parameter inference accuracy and robustness with respect to noise. Unlike in [3], all hyperparameters that control the smoothness of the interpolants are consistently inferred in the framework of nonparametric Bayesian statistics, which dispenses with the need to use heuristics and approximations in the configuration of the interpolation function.

We further the work of [4] in two respects. Firstly, we combine adaptive gradient matching using GPs with a parallel tempering scheme for the parameter that controls the mismatch between the gradients. This is conceptually different from the inference paradigm of the mismatch parameter that Dondelinger et al. [4] uses. Ideally, if the ODEs provide a correct mathematical description of the system, there should be no difference between the gradients of the interpolant and those predicted from the ODEs. However, in practice, forcing the gradients to be equal is likely to cause parameter inference methods to converge to a local optimum of the likelihood. Forcing the gradients to immediately be the same would restrict the inference procedure to a section of the likelihood corresponding to parameters that agree with the gradient match. However, there is no guarantee that these parameters are suitable for the data, see [5] for details. A parallel tempering scheme is the natural way to deal with such local optima, as opposed to inferring the degree of mismatch, since different tempering levels correspond to different strengths of penalising the mismatch between the gradients.Campbell and Steele [5] explore a parallel tempering scheme, but in order to get an understanding as to how well utilising this scheme improves inference, the rest of the set-up should be as similar as possible. Hence, comparing the results directly to the GP approach in [4], won’t provide us with this understanding, since the approach in [5] uses a different methodological paradigm. In this paper, we present a comparative assessment of parallel tempering versus inference in the context of gradient matching for the same modelling framework, i.e. without any confounding influence from the modelling choice. Secondly, we compare the approach of Bayesian inference using GPs with a variety of other methodological paradigms, within the specific context of comparing the gradients from the interpolant to the gradients from the ODEs, which is highly relevant to current computational systems biology.

We test the methods on two benchmark ODE systems: the Fitz–Hugh Nagumo system [which can model the behaviour of cardiac conditions such as: electrical excitation-conduction [6]; cardiac action potentials [7], and arrhythmias [8], as well as neurodegenerative diseases ([9, 10])], and a protein signalling transduction pathway [where cell signalling pathways can model cancers [11] and neurodegenerative diseases such as: Alzheimer’s disease; Parkinson’s disease, and amyotrophic lateral sclerosis (ALS) [12]], systems that are highly relevant to current biomedical engineering.

This paper is an extended version of [13] and includes a full description of the RKHS method in [14], as well as a series of comparative simulation studies using this method on the Fitz–Hugh Nagumo system, under different observational noise scenarios, and a protein signalling transduction pathway. The description of the methodology in [13] and [14] is also outlined in the review article [15], included here with permission from the authors.

## Methods

### Adaptive gradient matching with Gaussian processes

Consider a set of T arbitrary timepoints $t_1< \dots< t_i< \dots < t_T$, and noisy observations $\mathbf Y = (\mathbf y (t_1),...,\mathbf y (t_T))$, where $\mathbf y (t_i) = \mathbf x (t_i) + \varvec{\epsilon }(t_i)$, N = dim$\left( \mathbf x (t_i)\right)$, $\mathbf X = (\mathbf x (t_1),...,\mathbf x (t_T))$, $\mathbf y (t_i)$ is the data vector of the observations of all species concentrations at time $t_i$, $\mathbf x (t_i)$ is the vector of the concentrations of all species at time $t_i$, $\mathbf y _s$ is the data vector of the observations of species concentrations $s$ at all timepoints, $\mathbf x _s$ is the vector of concentrations of species $s$ at all timepoints, $y_s(t_i)$ is the observed datapoint of the concentration of species $s$ at time $t_i$, $x_s(t_i)$ is the concentration of species $s$ at time $t_i$ and $\varvec{\epsilon }$ is multivariate Gaussian noise, $\varvec{\epsilon } \sim N(\mathbf 0 ,\sigma ^2_s\mathbf I )$.

The time-dependent signals of the system can be described by ordinary differential equations

2 $$\begin{aligned} \dot{\mathbf{x }}_s= \frac{d\mathbf x _s}{dt_i} = f_s(\mathbf X ,\varvec{\theta }_s,\mathbf t ), \end{aligned}$$

which can be represented in scalar form as

3 $$\begin{aligned} \dot{x}_s(t_i) = \frac{dx_s(t_i)}{dt_i} = f_s(\mathbf x (t_i),\varvec{\theta }_s,t_i), \end{aligned}$$

where $f_s(\mathbf t ) = \left( f_s(t_1),\dots ,f_s(t_T)\right) ^{\mathsf{T}}$ and $\dot{\mathbf{x }}_s$ is the vector containing the gradients from the ODEs for species $s$ at all timepoints.

Then,

4 $$\begin{aligned} p(\mathbf Y |\mathbf X ,\varvec{\sigma }^2) = \prod \limits _s\prod \limits _t N(y_s(t_i)|x_s(t_i),\sigma ^2_s), \end{aligned}$$

where the dimension of the matrices $\mathbf X$ and $\mathbf Y$ are N by T. We take the approach in [1], and place a GP prior on $\mathbf x _s$,

5 $$\begin{aligned} p(\mathbf x _s|\varvec{\phi }_s,\varvec{\eta }) = N(\mathbf x _s|\varvec{\phi }_s,\mathbf K _{\eta _s}), \end{aligned}$$

where $\varvec{\phi }_s$ is a mean vector, which for simplicity we set as the sample mean, and $\mathbf K _{\eta _s}$ is a positive definite matrix of covariance functions with hyperparameters $\eta _s$. Differentiation is a linear operation, and therefore a GP is closed under differentiation ([16, 17]), meaning that the joint prior distribution of the concentrations of the species $\mathbf x _{s}$ and their time derivatives $\dot{\mathbf{x }}_{s}$ is multivariate Gaussian with mean $(\varvec{\phi }_{s},\mathbf 0 )^{\mathsf{T}}$ and covariance functions

6 $$\begin{aligned} cov[x_s(t_i),x_s(t_j)] = K_{\eta _s}(t_i,t_j), \end{aligned}$$

7 $$\begin{aligned} cov[\dot{x}_s(t_i),x_s(t_j)] = \frac{\partial K_{\eta _s}(t_i,t_j)}{\partial t_i} \; := \; K_{\eta _s}'(t_i,t_j), \end{aligned}$$

8 $$\begin{aligned} cov[x_s(t_i),\dot{x}_s(t_j)] = \frac{\partial K_{\eta _s}(t_i,t_j)}{\partial t_j} \; := \; 'K_{\eta _s}(t_i,t_j), \end{aligned}$$

9 $$\begin{aligned} cov[\dot{x}_s(t_i),\dot{x}_s(t_j)] = \frac{\partial ^2 K_{\eta _s}(t_i,t_j)}{\partial t_i \partial t_j}\; := \; K_{\eta _s}''(t_i,t_j), \end{aligned}$$

where $K_{\eta _s}(t_i,t_j)$ are the components of the covariance matrix $\mathbf K _{\eta _s}$. The conditional distribution for the state derivatives is obtained using elementary transformations of Gaussian distributions (see page 87 of [18] for details), yielding

10 $$\begin{aligned} p(\dot{\mathbf{x }}_s|\mathbf x _s,\varvec{\phi }_s,\varvec{\eta }_s) = N(\varvec{\mu }_s,\mathbf A _s), \end{aligned}$$

where

11 $$\begin{aligned} {\varvec{\mu }}_{s} = {{} \mathbf '\mathbf K _{\eta _s}}\mathbf{K _{\eta _s}}^{-1} (\mathbf x _{s}-\varvec{\phi }_{s}) \text { and } \mathbf A _{s} = \mathbf{K ''_{\eta _s}}- {{} \mathbf '\mathbf K _{\eta _s}}\mathbf{K _{\eta _s}}^{-1} \mathbf{K '_{\eta _s}}. \end{aligned}$$

We assume additive Gaussian noise with a state-specific error variance $\gamma _s$, and so, from Eq. (2), we get

12 $$\begin{aligned} p(\dot{\mathbf{x }}_s|\mathbf X ,\varvec{\theta }_s,\gamma _s) = N(f_s(\mathbf X ,\varvec{\theta }_s,\mathbf t ),\gamma _s\mathbf I ). \end{aligned}$$

Using a product of experts approach, Calderhead et al. [1], Dondelinger et al. [4], link the interpolant in Eq. (10) with the ODE model in Eq. (12), giving us the following distribution

13 $$\begin{aligned} \begin{array}{lll} p(\dot{\mathbf{x }}_s|\mathbf X ,\varvec{\theta }_s,\varvec{\phi }_s,\varvec{\eta }_s,\gamma _s) &{}\propto &{} p(\dot{\mathbf{x }}_s|\mathbf x _s,\varvec{\phi }_s,\varvec{\eta }_s)p(\dot{\mathbf{x }}_s|\mathbf X ,\varvec{\theta }_s,\gamma _s) \\ &{}=&{} N(\varvec{\mu }_s,\mathbf A _s)N(f_s(\mathbf X ,\varvec{\theta }_s,\mathbf t ),\gamma _s\mathbf I ). \end{array} \end{aligned}$$

Therefore, the joint distribution is

14 $$\begin{aligned} p(\dot{\mathbf{X }},\mathbf X ,\varvec{\theta },\varvec{\phi },\varvec{\eta },\varvec{\gamma }) = p(\varvec{\theta })p(\varvec{\eta })p(\varvec{\gamma })\prod \limits _s p(\dot{\mathbf{x }}_s|\mathbf X ,\varvec{\theta }_s,\varvec{\phi }_s,\varvec{\eta }_s,\gamma _s)p(\mathbf x _s|\varvec{\eta }_s), \end{aligned}$$

where $\varvec{\gamma }$ is the vector which contains all the gradient mismatch parameters and $p(\varvec{\theta }), p(\varvec{\eta }), p(\varvec{\gamma })$ are the prior distributions over the respective parameters. Dondelinger et al. [4] show that the marginalisation over the state derivatives yields a closed form solution to

15 $$\begin{aligned} p(\mathbf X ,\varvec{\theta },\varvec{\phi },\varvec{\eta },\varvec{\gamma }) = \int p(\dot{\mathbf{X }},\mathbf X ,\varvec{\theta },\varvec{\phi },\varvec{\eta },\varvec{\gamma })d\dot{\mathbf{X }}. \end{aligned}$$

Using the noise model in Eq. (4) and the closed form solution to Eq. (15), our full joint distribution becomes

16 $$\begin{aligned} p(\mathbf Y ,\mathbf X ,\varvec{\theta },\varvec{\phi },\varvec{\eta },\varvec{\gamma },\varvec{\sigma }^2) = p(\mathbf Y |\mathbf X ,\varvec{\sigma }^2)p(\mathbf X |\varvec{\theta },\varvec{\phi },\varvec{\eta },\varvec{\gamma }) p(\varvec{\theta })p(\varvec{\eta })p(\varvec{\gamma })p(\varvec{\sigma }^2), \end{aligned}$$

where $p(\varvec{\sigma }^2)$ is the prior over the variance of the observational error. The work in [4] shows

17 $$\begin{aligned} p(\mathbf X |\varvec{\theta },\varvec{\phi },\varvec{\eta },\varvec{\gamma }) \propto \frac{1}{C} \exp \left[ -\frac{1}{2} \sum _{s}\left( \mathbf x _s^T\mathbf K _{\eta _s} \mathbf x _s+ (\mathbf f _s-\varvec{\mu }_s)^{T}(\mathbf A _s+ \gamma _s\mathbf I )^{-1}(\mathbf f _s-\varvec{\mu }_s)\right) \right] , \end{aligned}$$

where $C = \prod _s|2\pi (\mathbf A _s+ \gamma _s\mathbf I )|^{\frac{1}{2}}$ and $\mathbf f _s$ is the vector containing the ODE predicted gradients for species $s$. Sampling is conducted using MCMC and the whitening approach of [19] is used to efficiently sample in the joint space of latent variables $\mathbf X$ and GP hyperparameters $\varvec{\eta }$.

*Parallel tempering:* Consider a series of “temperatures”, $0 = \alpha ^{(1)}<\cdots< \alpha ^{(M)} = 1$ and a power posterior distribution of our ODE parameters ([20])

18 $$\begin{aligned} p_{\alpha ^{(i)}}(\varvec{\theta }^{(i)}|\mathbf y ) \propto p(\varvec{\theta }^{(i)})p(\mathbf y |\varvec{\theta }^{(i)})^{\alpha ^{(i)}}. \end{aligned}$$

It is clear that Eq. (18) becomes the prior for $\alpha ^{(i)}$ = 0 and is the posterior when $\alpha ^{(i)}$ = 1. For $0< \alpha ^{(i)} < 1$ we get a distribution between our prior and posterior. The M $\alpha ^{(i)}$s in Eq. (18) are annealed likelihoods that are used as the target densities of parallel MCMC chains ([5]). At each MCMC step, all “temperature” chains independently perform a metropolis-hastings step to update $\varvec{\theta }^{(i)}$, the parameter vector associated with temperature $\alpha ^{(i)}$

19 $$\begin{aligned} p_{\text {move}} = \text {min}\left( 1,\frac{p\left( \mathbf y |\varvec{\theta }^{\text {prop}(i)}\right) ^{\alpha ^{(i)}}p\left( \varvec{\theta }^{\text {prop}(i)}\right) q\left( \varvec{\theta }^{\text {curr}(i)}|\varvec{\theta }^{\text {prop}(i)} \right) }{p\left( \mathbf y |\varvec{\theta }^{\text {curr}(i)}\right) ^{\alpha ^{(i)}}p\left( \varvec{\theta }^{\text {curr}(i)}\right) q\left( \varvec{\theta }^{\text {prop}(i)}|\varvec{\theta }^{\text {curr}(i)} \right) } \right) , \end{aligned}$$

where *q*( ) represents the proposal distribution and the superscripts “prop” and “curr” indicate whether the algorithm is being evaluated at the proposed or current state, respectively. At each MCMC step, two chains are randomly selected (uniformly) and the corresponding parameters are proposed to swap between them. This proposal has acceptance probability

20 $$\begin{aligned} p_{\text {swap}} = \mathrm {min}\left( 1,\frac{p_{\alpha ^{(j)}}(\varvec{\theta }^{(i)}|\mathbf y )p_{\alpha ^{(i)}}(\varvec{\theta }^{(j)}|\mathbf y )}{p_{\alpha ^{(i)}}(\varvec{\theta }^{(i)}|\mathbf y )p_{\alpha ^{(j)}}(\varvec{\theta }^{(j)}|\mathbf y )}\right) . \end{aligned}$$

The method we develop in this paper focuses on the intrinsic slack parameter $\gamma _s$ (see Eq. 12), which theoretically should be $\gamma _s=0$, since this corresponds to no mismatch between the gradients. In practice, to prevent the inference scheme from getting stuck in sub-optimal states, it is allowed to take on larger values $\gamma _s> 0$. However, rather than inferring $\gamma _s$ like a model parameter, as Dondelinger et al. [4] do, other authors (e.g. [5]) state that $\gamma _s$ should be gradually set to zero, since values closer to zero force the gradients to be more similar to one another and allow the interpolants to be informed by the ODEs. It is possible to abruptly set the values to zero, rather than gradually, however this is likely to cause the parameter inference techniques to converge to a local optimum of the likelihood. Hence, we combine the gradient matching with GPs approach in [4] with the tempering approach in [5] and temper this parameter to zero.

Prior to the parameter inference, we choose values of $\gamma _s$ and assign them to the variance parameter in Eq. (12) for each “temperature” $\alpha ^{(i)}$, such that chains closer to the prior ($\alpha ^{(i)}$ values closer to 0) allow the gradients from the interpolant to have more freedom to deviate from those predicted by the ODEs (which corresponds to larger $\gamma _s$ values), chains closer to the posterior ($\alpha ^{(i)}$ values closer to 1) more closely match the gradients (corresponding to smaller $\gamma _s$ values), and for the chain corresponding to $\alpha ^{(M)} = 1$, we want the mismatch to be approximately zero ($\gamma _s\approx 0$). Since $\gamma _s$ corresponds to the variance of our species-specific error (see Eq. 12), as $\gamma _s\rightarrow$ 0, we have less difference between the gradients, and as $\gamma _s$ gets larger, the gradients have more freedom to deviate from one another. Hence, we temper $\gamma _s$ towards zero. Now, each $\alpha ^{(i)}$ chain in Eq. (18) has a $\gamma _{s}^{(i)}$ (where the superscript $(i)$ indicates the gradient mismatch parameter associated with “temperature” $\alpha ^{(i)}$) fixed in place for the strength of the gradient mismatch. The specific schedules of the gradient mismatch parameter are included in Table 4.

### Reproducing kernel Hilbert space

Reproducing kernel Hilbert spaces (RKHS) allow for any function defined in an RKHS to be written as a linear combination of the kernel function evaluated at the training points. This provides a computationally fast process for interpolation. The objective function is expressed as

21 $$\begin{aligned} J(\mathbf c ) = \frac{1}{2\sigma ^2}|\mathbf y -\mathbf K {} \mathbf c |^2 + \frac{1}{2}{} \mathbf c ^{\mathsf{T}}{} \mathbf K {} \mathbf c , \end{aligned}$$

where $\mathbf y$ denotes the data, $\mathbf K$ is a matrix of kernel elements for all combinations of observed timepoints and $\mathbf c$ is a vector of coefficients. Minimising with respect to $\mathbf c$ gives us

22 $$\begin{aligned} \hat{\mathbf{c }} = (\mathbf K + \sigma ^2\mathbf I )^{-1}{} \mathbf y . \end{aligned}$$

Hence,

23 $$\begin{aligned} \hat{f}({t}_*) = \sum _{i=1}^{N}\hat{c}_ik({t}_*,{t}_i) = \mathbf k ^{\mathsf{T}}_{*}(\mathbf K + \sigma ^2\mathbf I )^{-1}{} \mathbf y , \end{aligned}$$

where ${t}_*$ is the timepoint at which one wants to make predictions and $\mathbf k _*$ is the vector of kernel elements for all combinations of $t_*$ and $t_i$. Note that this form is the same form as a posterior mean of a GP predictive distribution. For more details on RKHS, see [21].

### Penalised likelihood with RKHS

The aim of González et al. [14] is to create a penalised likelihood function that incorporates the information of the ODEs, then, using the properties of reproducing kernel Hilbert spaces, perform parameter estimation in a computationally fast manner. González et al. [14] consider ODEs of the form

24 $$\begin{aligned} \dot{\mathbf{x }}_s= g_s(\mathbf X ,\varvec{\rho }_s,\mathbf t ) - \delta _s\mathbf x _s, \end{aligned}$$

which can be represented in scalar form as

25 $$\begin{aligned} \dot{x}_s(t_i) = g_s(\mathbf x (t_i),\varvec{\rho }_s,t_i) - \delta _sx_s(t_i), \end{aligned}$$

where $\mathbf x _s$ is the vector of concentrations for species $s$, $\delta _s$ is the degradation rate of the concentrations for species $s$, $\varvec{\rho }_s$ is a parameter vector for species $s$ and $g_s(\mathbf t ) = \left( g_s(t_1),\dots ,g_s(t_T)\right) ^{\mathsf{T}}$. It is important to realise the difference between Eqs. (1) and (24). Whereas in Eq. (1), all parameter terms are included in the function $f_s()$, Eq. (24) considers the linear decay term separate to the rest of the ODE function $g_s(\mathbf X ,\varvec{\rho }_s,\mathbf t )$. Now consider a differencing matrix $\mathbf D$, where

26 $$\begin{aligned} \mathbf D = \Delta \left[ \begin{array}{llllll} -1 &{} 1 &{} 0 &{} \dots &{} \dots &{} 0\\ -1 &{} 0 &{} 1 &{} 0 &{} \dots &{} 0\\ 0 &{} -1 &{} \ddots &{} 1 &{} \ddots &{} \vdots \\ \vdots &{} \ddots &{} \ddots &{} \ddots &{} \ddots &{} \vdots \\ \vdots &{} \ddots &{} \ddots &{} \ddots &{} \ddots &{} \vdots \\ 0&{} \dots &{} \dots &{} \dots &{} -1&{} 1 \end{array}\right] , \end{aligned}$$

and $\Delta = \mathrm {diag}\left( \frac{1}{t_2-t_1},\frac{1}{t_3-t_1},\frac{1}{t_4-t_2},\dots ,\frac{1}{t_T-t_{T-2}},\frac{1}{t_T-t_{T-1}} \right)$. Equation (24) can then be approximated as

27 $$\begin{aligned} \mathbf D {} \mathbf x _s= g_s(\mathbf X ,\varvec{\rho }_s,\mathbf t ) - \delta \mathbf x _s. \end{aligned}$$

To make it clear how $\mathbf D {} \mathbf x _s$ is computed, as an example let us consider $\mathbf x _s= \left( x(t_1),\dots ,x(t_5) \right) ^{\mathsf{T}}$ and $\mathbf t = \left( 3,4,5,6,7 \right) ^{\mathsf{T}}$.

Then

28 $$\begin{aligned} \begin{array}{ll} \mathbf D {} \mathbf x _s = \left[ \begin{array}{lllll} \frac{1}{4-3}&{} &{} \\ &{}\frac{1}{5-3}&{} &{} &{}\\ &{} &{} \frac{1}{6-4} &{} &{}\\ &{} &{} &{} \frac{1}{7-5}&{}\\ &{} &{} &{} &{}\frac{1}{7-6}\end{array}\right] \left[ \begin{array}{lllll} -1&{}1&{}0&{}0&{}0\\ -1&{}0&{}1&{}0&{}0\\ 0&{}-1&{}0&{}1&{}0\\ 0&{}0&{}-1&{}0&{}1\\ 0&{}0&{}0&{}-1&{}1 \end{array}\right] \left[ \begin{array}{l} x(3)\\ x(4)\\ x(5)\\ x(6)\\ x(7)\end{array}\right] \\ \quad \quad = \left[ \begin{array}{lllll} \frac{-x(3)+x(4)}{1},&{}\frac{-x(3)+x(5)}{2},&{}\frac{-x(4)+x(6)}{2},&{}\frac{-x(5)+x(7)}{2},&{}\frac{-x(6)+x(7)}{1} \end{array}\right] ^\mathsf{T}. \end{array} \end{aligned}$$

Now denote $\mathbf R = \mathbf D +\delta _s\mathbf I$ (where $\mathbf I$ is the identity matrix). This gives us the following penalty to be incorporated into the likelihood term:

29 $$\begin{aligned} \varvec{\Omega }(\mathbf{x _s}) = ||\mathbf R {} \mathbf x _s- g_i(\mathbf X ,\varvec{\rho }_s,\mathbf t )||^2. \end{aligned}$$

From Eq. (27), we can see that $\mathbf R {} \mathbf x _s- g_s(\mathbf X ,\varvec{\rho }_s,\mathbf t ) = 0$. However, since $\mathbf x _s= \mathbf 0$ does not necessarily imply that $\varvec{\Omega }(\mathbf x _s)=0$, Eq. (29) cannot be expressed as a norm of $\mathbf x _s$ within the RKHS framework. In order to make them compatible, the authors transform the state variables $\mathbf x _s$ (and subsequently $\mathbf y _s$). Instead, consider

30 $$\begin{aligned} \tilde{\mathbf{x }}_s= \mathbf x _s- \mathbf R ^{-1}g_s(\mathbf X ,\varvec{\rho }_s,\mathbf t ). \end{aligned}$$

By multiplying both sides of Eq. (30) by $\mathbf R$ and taking squared norms we get the exact form of Eq. (29) ($||\mathbf R \tilde{\mathbf{x }}_s||^2 = ||\mathbf R {} \mathbf x _s- g_s(\mathbf X ,\varvec{\rho }_s,\mathbf t )||^2$). Similarly, the data are transformed

31 $$\begin{aligned} \tilde{\mathbf{y }}_s= \mathbf y _s- \mathbf R ^{-1}g_s(\mathbf X ,\varvec{\rho }_s,\mathbf t ), \end{aligned}$$

in order to correspond with the transformed states $\tilde{\mathbf{x }}_s$. The penalty function in Eq. (29) is now

32 $$\begin{aligned} \varvec{\Omega }(\tilde{\mathbf{x }}_s) = ||\mathbf R \tilde{\mathbf{x }}_s||^2 = \langle \mathbf R \tilde{\mathbf{x }}_s,\mathbf R \tilde{\mathbf{x }}_s\rangle = \tilde{\mathbf{x }}_s^{\mathsf{T}}{} \mathbf R ^{\mathsf{T}}{} \mathbf R \tilde{\mathbf{x }}_s. \end{aligned}$$

Equation (32) is now a proper norm, since when $\tilde{\mathbf{x }}_s=\mathbf 0$, this implies $\varvec{\Omega }(\tilde{\mathbf{x }}_s) = 0$. Denote $\mathbf K = (\mathbf R ^{\mathsf{T}}{} \mathbf R )^{-1}$. $\mathbf K$ is a matrix of kernel elements which define a unique RKHS. Hence,

33 $$\begin{aligned} \varvec{\Omega }(\tilde{\mathbf{x }}_s) = ||\tilde{\mathbf{x }}_s||_H^2 = \mathbf c ^{\mathsf{T}}{} \mathbf K {} \mathbf c , \end{aligned}$$

(where Eq. (33) is used as the term in the far right of Eq. (21) and $\mathbf c$ is given in Eq. (22)). We are able to obtain closed form expressions for the transformed state variables by using Eqs. (22) and (23) (the original expressions can be recovered using Eq. 30)

34 $$\begin{aligned} \tilde{\mathbf{x }}_s= \mathbf K (\mathbf K +2\lambda _s\varvec{\Sigma })^{-1}\tilde{\mathbf{y }}_s, \end{aligned}$$

where $\varvec{\Sigma }$ is the covariance matrix of the data (generalising Eq. 22, since the observational error of our data may not be independent between species) and $\lambda _s$ is a penalty parameter.

In the case of homogeneous ODEs, where $g_s()=0$, a kernel in a Hillbert space can be constructed using the Green’s function of the linear operator $\mathbf R$. $\mathbf K$ is the Green’s function of $\mathbf R ^\mathsf{T}{} \mathbf R$, where $\mathbf R ^\mathsf{T}$ is the adjoint operator of $\mathbf R$. Aronszajin et al. [22] show $||\mathbf R \tilde{\mathbf{x }}_s||^2_{L^2} = ||\tilde{\mathbf{x }}_s||^2_{H_\mathbf K }=\Omega (\tilde{\mathbf{x }}_s)$. Since the analytical form of Green functions of $\mathbf R ^\mathsf{T}{} \mathbf R$ is not available, the differential operator is approximated with the difference operator ($\mathbf D$). In the non-homogeneous ODE system, the model is linearised by feeding surrogate $\hat{\mathbf{x }}_s$ (using spline interpolation, in this case) into $g_s()$. $\Omega (\tilde{\mathbf{x }}_s)$ is still a valid RKHS norm for the transformed variable $\tilde{\mathbf{x }}_s$ defined in Eq. (30).

The penalised log-likelihood function is now expressed as

35 $$\begin{aligned} l(\varvec{\rho }_s,\delta _s,\varvec{\Sigma },\varvec{\alpha }_s,\mathbf c |\tilde{\mathbf{y }}_s) = \sum _{s=1}^{N}\left[ -\frac{1}{2}(\tilde{\mathbf{y }}_s-\tilde{\mathbf{x }}_s)^{\mathsf{T}}\varvec{\Sigma }^{-1}(\tilde{\mathbf{y }}_s-\tilde{\mathbf{x }}_s)-\frac{1}{2}ln|\varvec{\Sigma }|\right] - \sum _{s=1}^{N}\lambda _s\varvec{\Omega }(\tilde{\mathbf{x }}_s), \end{aligned}$$

where $\varvec{\alpha }_s$ is the vector containing the coefficients from the spline interpolant for species $s$. Parameter estimation using Eq. (35) can be carried out with standard non-linear optimisation algorithms such as quasi–Newton or conjugate gradients.

In the original paper of [14], the penalty parameter $\lambda _s$ is inferred using AIC. For a given value of $\lambda _s$, Eq. (35) is optimised to estimate the ODE parameters and subsequently the AIC score of the procedure is calculated. This is repeated for different $\lambda _s$ values and the $\lambda _s$ value corresponding to the smallest AIC score is chosen.

As well as using this approach for estimating $\lambda _s$, we found that using threefold cross validation, instead of AIC, provided more robust parameter estimation. We present the results from both schemes.

### Brief summary of methods

The detailed methodology we cover previously pertains to our new method and to the methods we were able to obtain the authors’ code for and adapt to many experimental settings. We were unable to obtain the authors’ code for the publication in Ramsay et al. [3]. The run times for the parallel tempering method of Campbell and Steele [5], using the authors’ own code, were excessive (typically in the order of days), and we were therefore unable to carry out an exhaustive exploration of the method. The methods of Ramsay et al. [3] and Campbell and Steele [5] are included as a benchmark comparison and so we leave it to the readers to refer to the original publications for full details. The following is a brief summary of all the methods we compare. Since many methods and settings are used in this paper for comparison purposes, abbreviations are used for ease of reading. Table 2 contains a key for those methods.

**Table 2:** Abbreviations of the methods used throughout this paper. Table reproduced from [13], with permission from Springer

Abbreviation	Method	Reference
C&S	Tempered mismatch parameter using splines-based smooth functional tempering	Campbell and Steele [5]
INF	Inference of the gradient mismatch parameter using GPs	Dondelinger et al. [4]
LB2	Tempered mismatch parameter using GPs in log base 2 increments	Our method
LB10	Tempered mismatch parameter using GPs in log base 10 increments	Our method
GON	Reproducing kernel Hilbert space and penalised likelihood. The penalty parameter is estimated using AIC	González et al. [14]
GON Cross	Reproducing kernel Hilbert space and penalised likelihood. The penalty parameter is estimated using 3-fold cross validation	González et al. [14]
RAM	Hierarchical 3 level regularisation approach using splines based interpolation	Ramsay et al. [3]

**C&S ** [5]: Parameter inference is carried out using adaptive gradient matching and tempering of the mismatch parameter. B-splines are used as the choice of interpolation scheme. **INF** [4]: This method conducts parameter inference through adaptive gradient matching using GPs. The penalty mismatch parameters $\varvec{\gamma }_s$ are inferred. **LB2**: This method conducts parameter inference through adaptive gradient matching using GPs. The penalty mismatch parameters $\varvec{\gamma }_s$ are tempered in log base 2 increments, see Table 4 for details. **LB10**: As with LB2, parameter inference is conducted through adaptive gradient matching using GPs, however, the penalty mismatch parameters $\varvec{\gamma }_s$ are tempered in log base 10 increments, see Table 4 for details. **GON** [14]: Parameter inference is conducted in a non-Bayesian fashion, implementing a reproducing kernel Hilbert space (RKHS) and penalised likelihood approach. Comparisons between RKHS and GPs have been previously explored conceptually (for example, see [21, 23]), and in this paper we analyse them empirically in the specific context of inference in ODEs. The RKHS method that incorporates the information from the ODEs in [14] obtains the ODE kernel using a differencing operator. AIC is used to estimate the penalty parameter $\lambda$. **GON Cross** [14]: The method is the same as **GON**, however, cross validation is used to estimate the penalty parameter $\lambda$, instead of AIC. **RAM** [3]: This technique uses a non-Bayesian optimisation process for parameter inference. The method penalises the difference between the gradients using splines and a hierarchical 3 level regularisation approach is used to set the tuning parameters (see [3] for details). Table 3 describes particular settings with some of the methods in Table 2. The ranges of the penalty parameters $\varvec{\gamma }_s$, for the LB2 and LB10 methods are given in Table 4. The increments are linear on the log scale. The M $\alpha _s$s from 0 to 1 are set by taking a series of M equally spaced values and raising them to the power 5, as described in [20].

**Table 3:** Particular settings of Campbell and Steele’s [5] method

Abbreviation	Definition	Details
10C	10 chains	When comparing our methods, it was of interest to see how the performance depended on the number of parallel MCMC chains, as originally the authors used 4 chains
Obs20	20 observations	Originally, the authors use 401 observations. We reduced this to a dataset size more usual with these types of experiments to observe the dependency of the methods on the amount of data
15K	15 knots	The method in C&S uses B-splines interpolation. We changed the original tuning parameters from the authors' paper to observe the sensitivity of the parameter estimation by these tuning parameters
P3	polynomial order 3 (cubic spline)	The original polynomial order is 5 and again, we wanted to observe the sensitivity of the parameter estimation by these tuning parameters

Table reproduced from [13], with permission from Springer

**Table 4:** Ranges of the penalty parameter $\varvec{\gamma }_s$ for LB2 and LB10

Method	Chains	Range of penalty $\varvec{\gamma }$
LB2	4	[1 , 0.125]
LB2	10	[1 , 0.00195]
LB10	4	[1 , 0.001]
LB10	10	$[1 , 1e^{-9}]$

In this paper $\varvec{\gamma }_s= \varvec{\gamma } \forall s$

Table reproduced from [13], with permission from Springer

## Data

*Fitz–Hugh Nagumo *([24, 25]): These equations originally were used to describe the voltage potential across the cell membrane of the axon of giant squid neurons. There are 3 parameters; $\alpha$, $\beta$ and $\psi$ and two “species”; Voltage (V) and Recovery variable (R). Species in [ ] denote the time-dependent concentration for that species:

36 $$\begin{aligned} \dot{[V]} = \psi ([V] - \frac{[V]^3}{3} + [R]) \end{aligned}$$

37 $$\begin{aligned} \dot{[R]} = -\frac{1}{\psi }([V] - \alpha + \beta *[R]) \end{aligned}$$

The Fitz–Hugh Nagumo equations are used in biomedical engineering to model features such as cardiac conditions (i.e. electrical excitation-conduction in cardiac tissue [6], cardiac action potentials [7] and arrhythmias [8]) and neurodegenerative diseases (Drosophila courtship can be modelled using these equations and used to screen genes linked to memory-deficiency and human neurodegeneration [9] and the system can also be used for diagnosing leprosy [10]).

*Protein signalling transduction pathway* [26]: These equations describe protein signalling transduction pathways in a signal transduction cascade [26], where the kinetic parameters control how quickly the proteins (“species”) convert to one another. There are 6 parameters ($k_1, k_2, k_3, k_4, V, K_m$) and 5 “species” (*S*, *dS*, *R*, *RS*, *Rpp*). The system describes the phosphorylation of a protein, $R \rightarrow Rpp$ (Eq. 42), catalysed by an enzyme *S*, via an active protein complex [*RS*, Eq. (41)], where the enzyme is subject to degradation [$S \rightarrow dS$, Eq. (39)]. The chemical kinetics are described by a combination of mass action kinetics [Eqs. (38), (39), (41)] and Michaelis–Menten kinetics [Eqs. (40), (42)]. Species in [ ] denote the time-dependent concentration for that species:

38 $$\begin{aligned} \dot{[S]} = -k_1*[S] - k_2*[S]*[R] + k_3*[RS] \end{aligned}$$

39 $$\begin{aligned} \dot{[dS]} = k_1*[S] \end{aligned}$$

40 $$\begin{aligned} \dot{[R]} = -k_2*[S]*[R] + k_3*[RS] + \frac{V*[Rpp]}{K_m + [Rpp]} \end{aligned}$$

41 $$\begin{aligned} \dot{[RS]} = k_2*[S]*[R] - k_3*[RS] -k_4*[RS] \end{aligned}$$

42 $$\begin{aligned} \dot{[Rpp]} = k_4*[RS] - \frac{V*[Rpp]}{K_m + [Rpp]} \end{aligned}$$

Cell signalling is a highly relevant topic in current biomedical engineering. The equations can model cancers [11] and neurodegenerative diseases that include Alzheimer’s disease, Parkinson’s disease and ALS [12].

These ODE systems give us benchmark data and produce periodic signals (in the Fitz–Hugh Nagumo system) and signals that make a transition to a stationary phase (protein signalling transduction pathway), which is representative of models in this area. Hence, we can assess the methods discussed in this paper on systems that are meaningful to the field of biomedical engineering.

## Simulation

We have compared the proposed GP tempering scheme with the alternative methods summarised in the "Methods" section. For the comparison to Ramsay et al. [3], we were unable to obtain the authors’ software and so we compared our results directly with the results from the original publications. Hence, we generated test data in the same manner as described by the authors and used them for the evaluation of our method. For the methods in Campbell and Steel [5], Dondelinger et al. [4] and González et al. [14], where we did receive the authors’ software, we repeated the evaluation twice, first on data equivalent to those used in the original publications, and again on new data generated with different (more realistic) parameter settings. For comparisons using the Fitz–Hugh Nagumo model, Eqs. (36) and (37), we used the ODE prior distributions in [5] and for comparisons using the protein signalling transduction pathway model, Eqs. (38–42), we used the parameter priors from [4]. This gave us priors that were motivated by the current literature. Our code is available upon request.

*Tempered mismatch parameter using splines-based smooth functional tempering (C&S)* [5]: The authors tested their method on the Fitz–Hugh Nagumo system, Eqs. (36) and (37), with the following parameter settings: $\alpha =0.2$, $\beta =0.2$ and $\psi =3$, starting from initial values of $(-1,1)$ for the two “species”. They generated 401 observations over the time course [0, 20] (producing 2 periods) and Gaussian noise with sd {0.5, 0.4} was used to corrupt each respective “species”. To infer the ODE parameters with their approach, the authors chose the following settings: B-splines of polynomial order 5 with 301 knots; 4 parallel tempering chains, gradient mismatch parameter schedules {10,100,1000,10000}; parameter prior distributions for the ODE parameters: $\alpha \sim N(0, 0.4^2)$, $\beta \sim N(0, 0.4^2)$ and $\psi \sim \chi ^2_2$.

As well as comparing our method with the results the authors had obtained with their original settings, we made the following modifications to test the robustness of their procedure. We reduced the number of observations from 401 to 20 over the time course [0, 10] (producing 1 period), which more closely reflects the amount of data typically available in current systems biology. In doing so, we also reduced the number of knots for the splines to 15 (preserving the same proportionality of knots to datapoints as before), and we tried a different polynomial order: 3 instead of 5. The method incurred high computational costs, (roughly $1 \frac{1}{2}$ weeks for a run), and so we could only repeat the inference on 3 independent data sets. The posterior samples were combined in order to approximately marginalise over datasets and thereby remove their potential particularities. For a fair comparison, we also ran our methods with 4 rather than the 10 chains that we used as default.

*Inference of the gradient mismatch parameter using GPs and adaptive gradient matching (INF)* [4]: We applied the method in the same way as described in the original publication of [4], using the authors’ software and selecting the same kernels and parameter/hyperparameter priors for the method proposed in the present paper. We generated data from the protein signal transduction pathway described in Eqs. (38–42), with the same settings as in [4]; initial values of the species: $(S=1, dS=0, R=1, RS=0, Rpp=0)$; ODE parameters: $(k_1=0.07, k_2=0.6, k_3=0.05, k_4=0.3, V=0.017, K_m=0.3)$; 15 timepoints producing one period: $\{0, 1, 2, 4, 5, 7, 10, 15, 20, 30, 40, 50, 60, 80, 100\}$. As in [4], we used multiplicative iid Gaussian noise of standard deviation $=0.1$ to corrupt the signals and reflect the noisy observations obtained in experiments. We chose the same gamma prior on the ODE parameters as used in [4], namely $\Gamma (4, 0.5)$, for Bayesian inference. For the GP, we used the same kernel they originally used; see further on for details. In addition to this ODE system, we also applied this method to the rest of the described set-ups.

*Reproducing kernel Hilbert space method (GON)* [14]: The authors tested their method on the Fitz–Hugh Nagumo data (Eqs. 36, 37) with the following settings; initial values of $(-1 , -1)$ and ODE parameters of $\alpha =0.2$; $\beta =0.2$ and $\psi =3$. The authors generated 50 datapoints over the time domain [0, 20] (producing 2 periods), with iid Gaussian noise (sd = 0.1) added to introduce error to the observations. 50 independent data sets were created in this way.

As well as comparing to the original publication set-up, we also tested the methods on a scenario with larger observational noise. We tested on 2 scenarios, when the signal to noise ratio was on average 10 for each species and when the average signal to noise ratio was 5. We used the average signal to noise ratio so that each species had the same observational error as one another. We reduced the dataset size to 25 timepoints over the time course [0, 10], producing 1 period, and show the results across 10 independent datasets.

To observe the variation between ODE models, we also ran the method on the protein signal transduction pathway in Eqs. (38–42). We generated data under the same settings as in [4]; ODE parameters: $(k_1=0.07, k_2=0.6, k_3=0.05, k_4=0.3, V=0.017, K_m=0.3)$; initial values of the species: $(S=1, dS=0, R=1, RS=0, Rpp=0)$; 15 timepoints covering one period: $\{0, 1, 2, 4, 5, 7, 10, 15, 20, 30, 40, 50, 60, 80, 100\}$. We examined 2 noise scenarios; when the average signal to noise ratio was 10, and when the average signal to noise ratio was 5. As opposed to the set-up in [4], we use additive Gaussian noise to corrupt the data, to correspond with the assumed noise model.

*Penalised splines and 2nd derivative penalty method (RAM) *[3]: González et al. [14] used the method of Ramsay et al. [3] to compare with their technique. We have used the results from the original publication of [14]. For fairness of comparison, our method was applied in the same way as with the set-up in [14].

*Choice of kernel*: For the GP, we need to choose a suitable kernel, which reflects our prior knowledge in function space. We considered two kernels in our study (to correspond with the authors’ set-ups), the radial basis function (RBF) kernel

43 $$\begin{aligned} k(t_i, t_j) \; =\; \sigma ^2_{\mathrm {RBF}}\exp (-\frac{(t_i-t_j)^2}{2l^2}) \end{aligned}$$

with hyperparameters $\sigma ^2_{\mathrm {RBF}}$ and $l^2$, and the sigmoid variance kernel

44 $$\begin{aligned} \small { \begin{array}{l} k(t_i, t_j) \; =\; \sigma ^2_{\mathrm {sig}}\arcsin \frac{a+(bt_it_j)}{\sqrt{(a+(bt_it_i)+1)(a+(bt_jt_j)+1)}} \end{array}} \end{aligned}$$

with hyperparameters $\sigma ^2_{\mathrm {sig}}$, *a* and *b* [23].

To initialise the hyperparameters, we fit a standard GP regression model (i.e. without information from the ODE) using maximum likelihood. We then checked to see whether the interpolant adequately represents our prior knowledge.

We found that the RBF kernel provided a good fit to the data for the data generated from the Fitz-Hugh Nagumo model. However, in confirmation of the findings in [4], we found that for the protein signalling transduction pathway, the non-stationary nature of the data is not represented properly with the RBF kernel, which is stationary [23]. As in [4], we used the sigmoid variance kernel, which is non-stationary [23] and found a considerable improvement to the fit to the data.

*Other settings*: We need to set the values for our variance mismatch parameter of the gradients, $\gamma _s$. Since studies that indicate reasonable values for our technique are limited (see [1, 20]), we used $Log_2$ and $Log_{10}$ increments with an initial start at 1. All parameters were initialised with a random draw from the respective priors (apart from GON, which did not use priors).

## Results

**Fig. 1:**
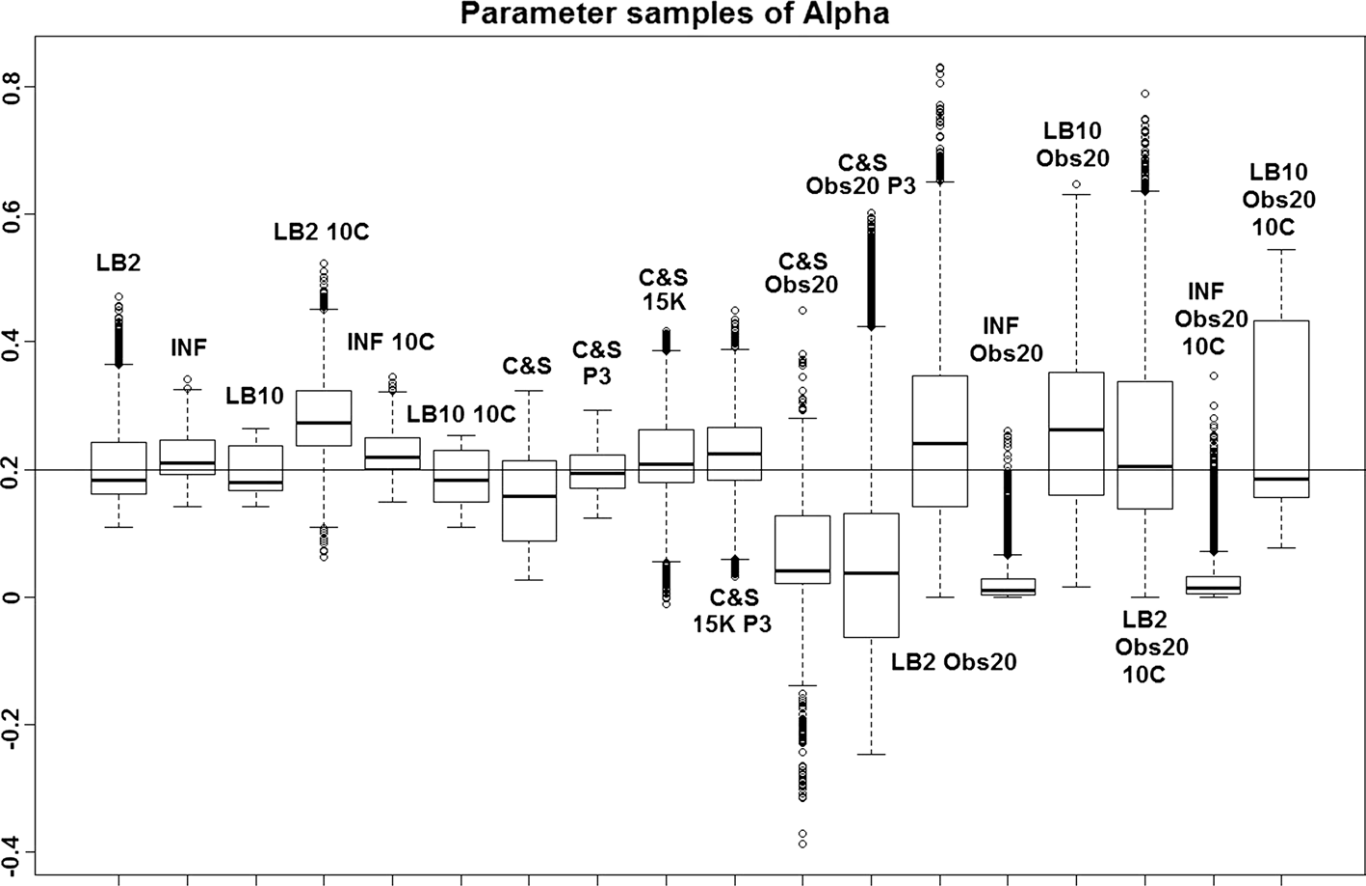
Average posterior distributions of parameter $\alpha$ from the Fitz–Hugh Nagumo model (Eq. 37) over 3 datasets. From* left to right*: LB2, INF, LB10, LB2 10C, INF 10C, LB10 10C, C&S, C&S P3, C&S 15K, C&S 15K P3, C&S Obs20, C&S Obs20 P3, LB2 Obs20, INF Obs20, LB10 Obs20, LB2 Obs20 10C, INF Obs20 10C and LB10 Obs20 10C. The* solid line* is the true parameter. For definitions, see Tables 2 and 3. Figure reproduced from [13], with permission from Springer

**Fig. 2:**
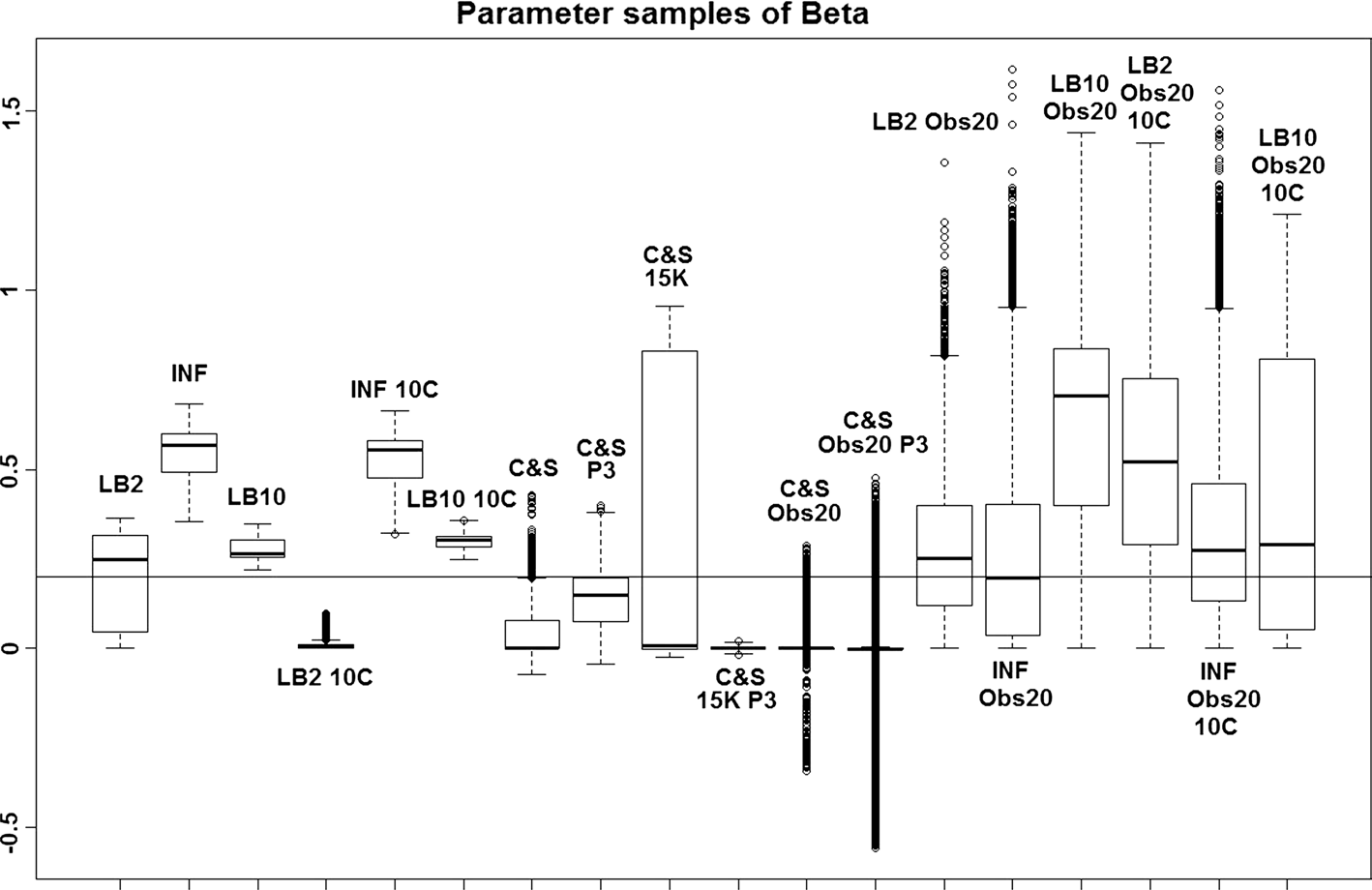
Average posterior distributions of parameter $\beta$ from the Fitz–Hugh Nagumo model (Eq. 37) over 3 datasets. From* left to right*: LB2, INF, LB10, LB2 10C, INF 10C, LB10 10C, C&S, C&S P3, C&S 15K, C&S 15K P3, C&S Obs20, C&S Obs20 P3, LB2 Obs20, INF Obs20, LB10 Obs20, LB2 Obs20 10C, INF Obs20 10C and LB10 Obs20 10C. The* solid line* is the true parameter. For definitions, see Tables 2 and 3. Figure reproduced from [13], with permission from Springer

**Fig. 3:**
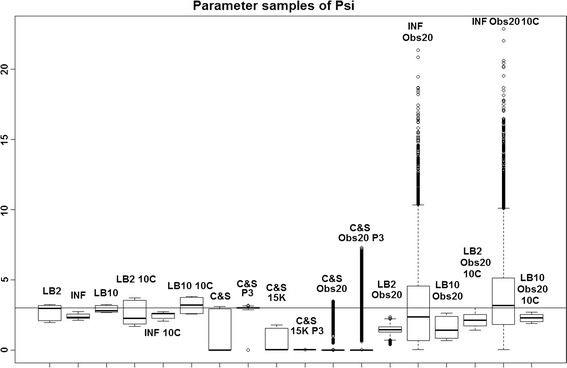
Average posterior distributions of parameter $\psi$ from the Fitz–Hugh Nagumo model (Eqs. 36, 37) over 3 datasets. From* left to right*: LB2, INF, LB10, LB2 10C, INF 10C, LB10 10C, C&S, C&S P3, C&S 15K, C&S 15K P3, C&S Obs20, C&S Obs20 P3, LB2 Obs20, INF Obs20, LB10 Obs20, LB2 Obs20 10C, INF Obs20 10C and LB10 Obs20 10C. The* solid line* is the true parameter. For definitions, see Tables 2 and 3. Figure reproduced from [13], with permission from Springer

*Tempered mismatch parameter using splines-based smooth functional tempering (C&S)* [5]: By examining Figs. 1, 2 and 3, we can see that the C&S method shows good performance over all parameters in the one case where the number of observations is 401, the number of knots is 301 and the polynomial order is 3 (cubic spline), since the bulk of the distributions of the sampled parameters surround the true parameters in Figs. 1 and 3 and are close to the true parameter in Fig. 2. These settings, however, require a great deal of “hand-tuning” or time expensive cross-validation and would be very difficult to set when using real data. We can observe the sensitivity of the method in the other set-ups, where the results are noticeably worse. An important point to note is when the dataset size was reduced, the cubic spline performed very badly. This lack of robustness makes these splines based methods very difficult to apply in practice. The INF, LB2 and LB10 methods consistently outperform the C&S method with distributions being closer to or overlapping the true parameters. On the set-up with 20 observations, for both 4 and 10 chains, the INF method produced largely different estimates across the datasets, as depicted by the wide boxplots and long tails.

**Fig. 4:**
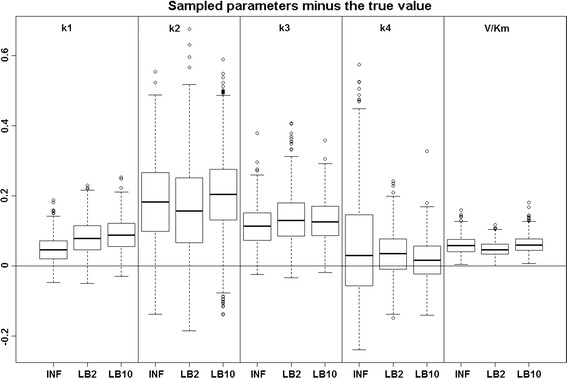
Results from the dataset that showed the average RMS of the posterior parameter samples minus the true values for the INF, LB2 and LB10 methods. The posterior distributions are of the sampled parameters from the protein signalling transduction pathway (Eqs. 38–42) minus the true value. The* horizontal line* shows zero difference. For definitions, see Tables 2 and 3. Figure reproduced from [13], with permission from Springer

*Inference of the gradient mismatch parameter using GPs, adaptive method (INF)* [4]: In order to see how the LB2 and LB10 tempering methods perform in comparison to the INF method, we can examine the results from the protein signalling transduction pathway (see Eqs. 38–42), as well as comparing how each method did in the other set-ups. Figure 4 shows the distributions of parameter estimates minus the true values for the protein signalling transduction pathway. After implementing the authors' code, we noticed that some of the MCMC simulations had not converged. In order to present a fair depiction of the methods’ performance, we show results from the dataset that produced the median performance. For each dataset the root mean square was calculated on the parameter samples minus the true values. The dataset that produced the median root mean square value is given.

We can see by examining Fig. 4, that for each parameter the methods are performing well, since the distributions are close to the true values. For this set-up, overall there does not appear to be a significant difference between the INF, LB2 and LB10 methods.

**Fig. 5:**
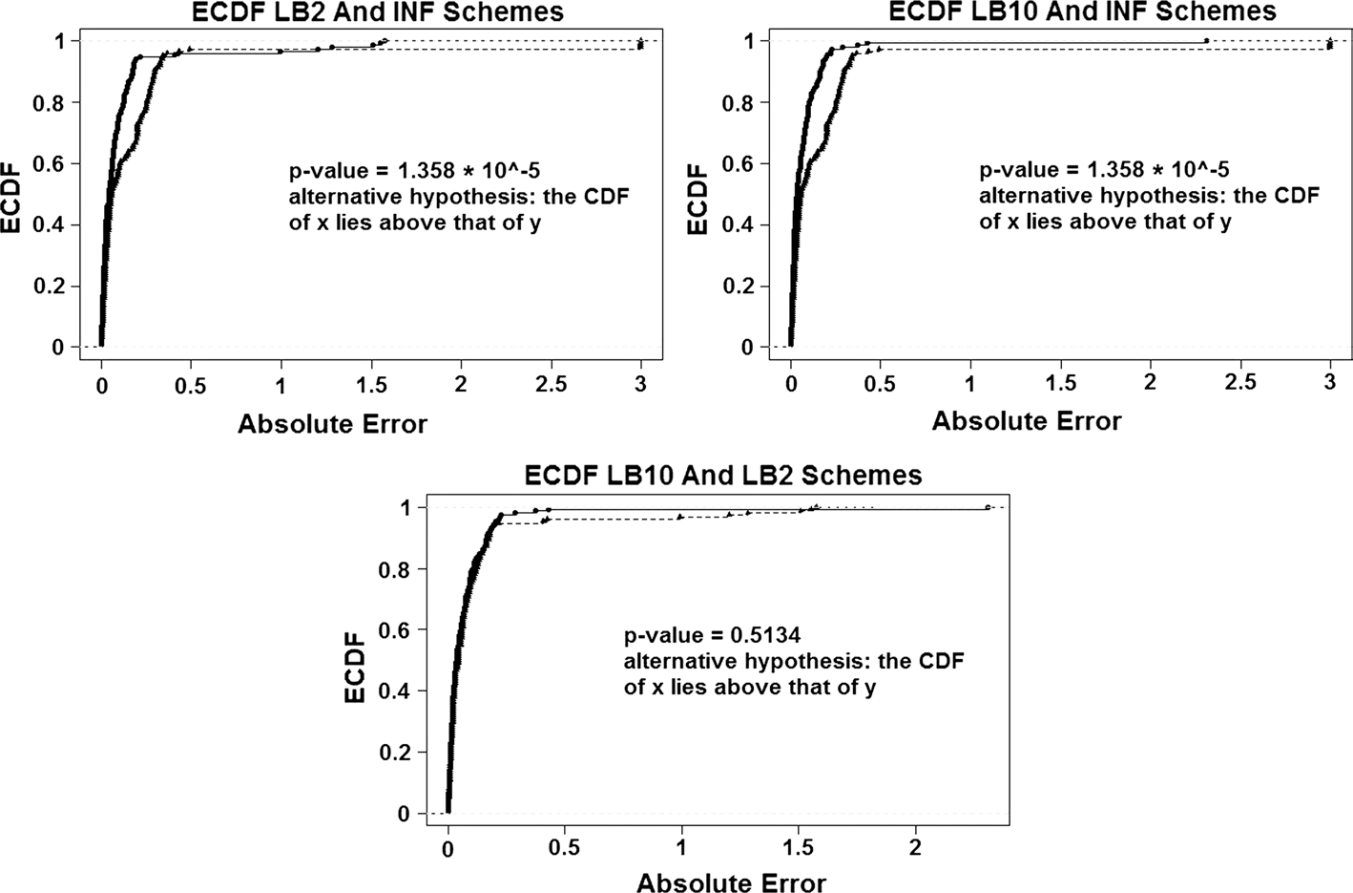
ECDFs of the absolute errors of the parameter estimation for the protein signalling transduction pathway (Eqs. 38-42).* Top left* —ECDFs for LB2 and INF,* top right*—ECDFs for LB10 and INF and* bottom*—ECDFs for LB10 and LB2. Included are the p-values for 2-sample, 1-sided Kolmogorov–Smirnov tests. For definitions, see Tables 2 and 3. Figure reproduced from [13], with permission from Springer

For the original set-up in [14], Fig. 5 shows the expected cumulative distribution functions (ECDFs) of the absolute errors of the parameter samples for the tempering and inference schemes. P-values for 2-sample, 1-sided Kolmogorov-Smirnov tests are given. Since the distributions are of the average error, if a distribution’s ECDF is significantly higher than another’s, this constitutes better parameter estimation. A higher curve means that there are more values located in the lower range of absolute error.

By examining Fig. 5 and using the standard significance level of 0.05 as a cut-off, we can see that the CDFs for LB2 and LB10 are significantly higher than those for INF. This means that the parameter estimates from the LB2 and LB10 methods are closer to the true parameters than the INF method, since we are dealing with absolute error. The LB2 and LB10 method show no significant difference to each other.

For the set-up in [5], Figs. 1, 2 and 3 show us that the LB2 and LB10 methods perform well across dataset size and over all the parameters, since most of the mass of the distributions surround or are situated close to the true parameters. One type of scheduling did not always outperform another, the LB2 does better than the LB10 for 4 parallel chains (distributions overlapping the true parameter for all three parameters) and the LB10 outperforms the LB2 for 10 parallel chains (distribution overlapping true parameter in Fig. 1, being closer to the true parameter in Fig. 2 and narrower and more symmetric around the true parameter in Fig. 3). The bulks of parameter sample distributions for the INF method are located close to the true parameters for all dataset sizes. However, the method produces less uncertainty at the expense of bias. When reducing the dataset size to 20 observations, for both 4 and 10 chains, the results deteriorate for the INF method and it is outperformed by the LB2 and LB10 methods.

**Fig. 6:**
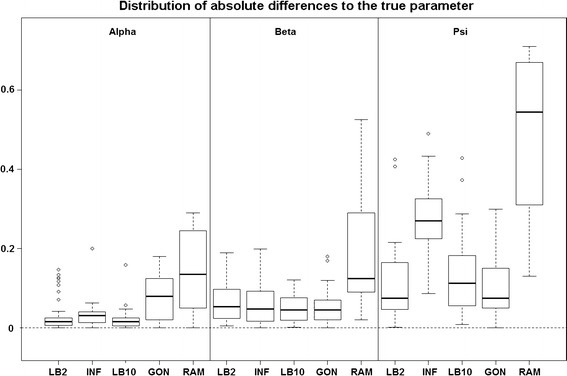
*Boxplots* of the distributions of the absolute differences of an estimate to the true parameter over 50 datasets. The three sections from* left to right* represent the parameters $\alpha$, $\beta$ and $\psi$ from the Fitz–Hugh Nagumo model (Eqs. 36, 37). Within each section, the* boxplots* from* left to right* are: LB2 method, INF method, LB10 method, GON method (*boxplot* reconstructed from [14]) and RAM method (*boxplot* reconstructed from [14]). For definitions, see Tables 2 and 3. Figure reproduced from [13], with permission from Springer

*Reproducing kernel Hilbert space (GON)* [14]* and Hierarchical regularisation splines based method (RAM)* [3]: For these sets of results, to assess the performance of the methods, we used the same criterion as in GON. For each parameter, the absolute value of the difference between an estimator and the true parameter ($|\hat{\theta }_i - \theta _i|$) was computed and the distribution across the datasets was examined. For the LB2, LB10 and INF methods, we used the median of the sampled parameters as an estimator, since it is a robust average. Examining Fig. 6, the LB2, LB10 and INF methods do as well as the GON method for 2 parameters (INF doing slightly worse for $\psi$) and outperform it for 1 parameter with the width of the distributions of the absolute distances to the true parameter roughly $\frac{1}{3}$ of the size. All methods outperform the RAM method.

**Fig. 7:**
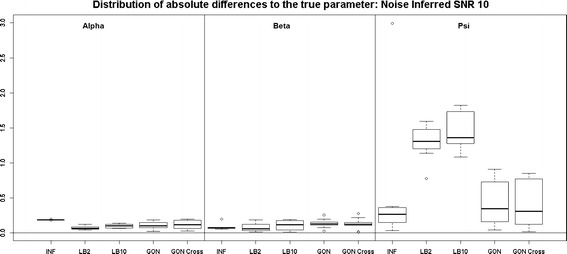
*Boxplots* of the distributions of the absolute differences of an estimate to the true parameter over 10 datasets. The three sections from* left to right* represent the parameters $\alpha$, $\beta$ and $\psi$ from the Fitz–Hugh Nagumo model (Eqs. 36, 37). Within each section, the* boxplots* from* left to right* are: LB2 method, INF method, LB10 method, GON method and GON method using cross validation for inferring the penalty parameter. The average signal to noise ratio for each “species” is 10. The standard deviation of the observational noise is inferred. For definitions, see Tables 2 and 3

**Fig. 8:**
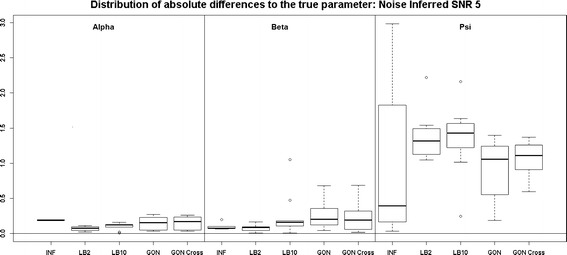
*Boxplots* of the distributions of the absolute differences of an estimate to the true parameter over 10 datasets. The three sections from* left to right* represent the parameters $\alpha$, $\beta$ and $\psi$ from the Fitz–Hugh Nagumo model (Eqs. 36, 37). Within each section, the* boxplots* from* left to right* are: LB2 method, INF method, LB10 method, GON method and GON method using cross validation for inferring the penalty parameter. The average signal to noise ratio for each “species” is 5. The standard deviation of the observational noise is inferred. For definitions, see Tables 2 and 3

Looking at Fig. 7, the performance of LB2 and LB10 is poorer for the 3$^{rd}$ parameter than the other methods by about 1 unit in absolute difference to the true parameter. When the noise is increased, Fig. 8, the GON and GON Cross methods are more robust in estimating the final parameter, where overall the average absolute error to the true parameter is about 0.5 smaller.

**Fig. 9:**
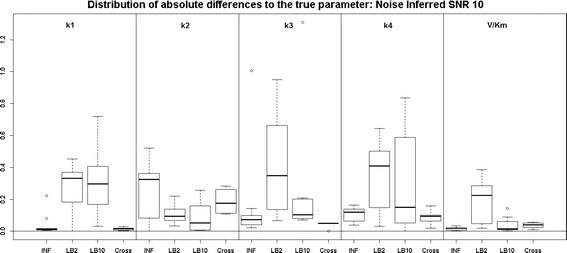
*Boxplots* of the distributions of the absolute differences of an estimate to the true parameter over 10 datasets. The 5 sections from* left to right* represent the parameters for the protein signalling transduction pathway, Eqs. (38–42). Within each section, the* boxplots* from* left to right* are: LB2 method, INF method, LB10 method and GON method using cross validation for inferring the penalty parameter. The average signal to noise ratio for each “species” is 10. The standard deviation of the observational noise is inferred. For definitions, see Tables 2 and 3

**Fig. 10:**
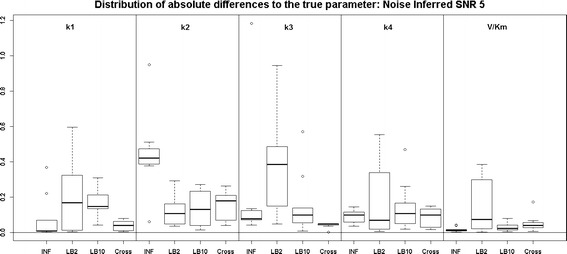
*Boxplots* of the distributions of the absolute differences of an estimate to the true parameter over 10 datasets. The 5 sections from* left to right *represent the parameters for the protein signalling transduction pathway, Eqs. (38–42). Within each section, the* boxplots* from* left to right* are: LB2 method, INF method, LB10 method and GON method using cross validation for inferring the penalty parameter. The average signal to noise ratio for each “species” is 5. The standard deviation of the observational noise is inferred. For definitions, see Tables 2 and 3

Examining the results for the protein signalling transduction pathway, Eqs. (38–42), in Figs. 9 and 10, we can see that the performance of INF, LB2 and LB10 vary in accuracy. The GON Cross method shows a more robust set of estimates, with results that are on average 0.2 units in absolute value closer to the true parameters. The GON method (which uses AIC to estimate the penalty parameter) was unable to optimise for this ODE system. Given certain values of $\lambda _s$, the optimiser of the log likelihood function tends to choose kernel parameters which make $(\mathbf K + \lambda _s\sigma _sI)$ non-invertible and computationally singular. In the cross validation scheme, all problematic $\lambda _s$s are rejected. We present the results for the GON Cross method only, for this ODE model.

We also present the root mean square (RMS) values in function space. Firstly, the signal was reconstructed with the sampled parameters, by numerically integrating the ODEs, and then the true signal was subtracted (signal created with true parameters and no observational noise added). The RMS was calculated on these residuals. It is important to assess the methods on this criterion as well as looking at the parameter uncertainty, as some parameters might only be weakly identifiable, corresponding to ridges in the likelihood landscape. In other words, large uncertainty in parameter estimates may not necessarily imply a poor performance by a method, if the reconstructed signals for all groups of sampled parameters were close to the truth.

**Fig. 11:**
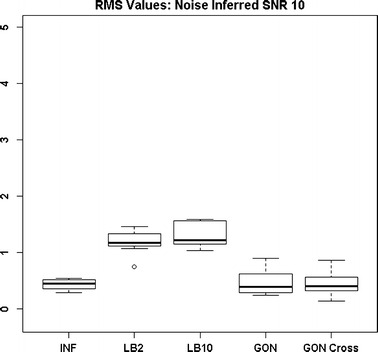
Distribution of RMS values in function space, calculated on the residuals of the true signal (signal produced with true parameters and no observational error) minus the signal produced with the estimate of the parameters, for the Fitz–Hugh Nagumo model (Eqs. 36, 37). Within each section, the* boxplots* from* left to right* are: LB2 method, INF method, LB10 method, GON method and GON method using cross validation for inferring the penalty parameter. The average signal to noise ratio for each “species” is 10. The standard deviation of the observational noise is inferred. For definitions, see Tables 2 and 3

**Fig. 12:**
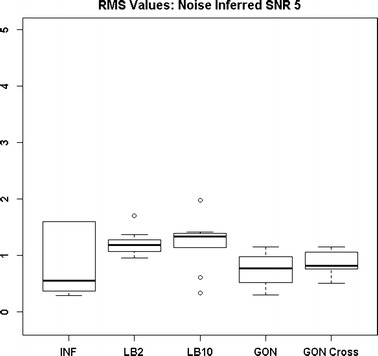
Distribution of RMS values in function space, calculated on the residuals of the true signal (signal produced with true parameters and no observational error) minus the signal produced with the estimate of the parameters, for the Fitz–Hugh Nagumo model (Eqs. 36, 37). Within each section, the* boxplots* from* left to right* are: LB2 method, INF method, LB10 method, GON method and GON method using cross validation for inferring the penalty parameter. The average signal to noise ratio for each “species” is 10. The standard deviation of the observational noise is inferred. For definitions, see Tables 2 and 3

By examining Fig. 11 we can see that the LB2 and LB10 methods perform poorer than the rest, with an average RMS value roughly 0.5 larger. In Fig. 12, the increased noise scenario, we can see that the LB2 and LB10 methods have an average RMS value about 0.5 units larger than the other methods.

**Fig. 13:**
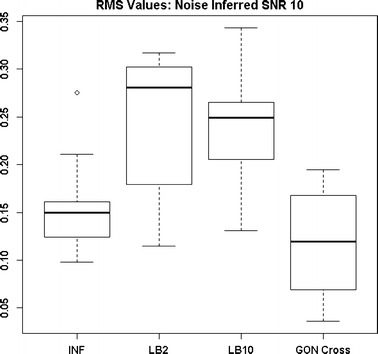
Distribution of RMS values in function space, calculated on the residuals of the true signal (signal produced with true parameters and no observational error) minus the signal produced with the estimate of the parameters, for the protein signalling transduction pathway (Eqs. 38–42). Within each section, the* boxplots* from* left to right* are: LB2 method, INF method, LB10 method and GON method using cross validation for inferring the penalty parameter. The average signal to noise ratio for each “species” is 10. The standard deviation of the observational noise is inferred. For definitions, see Tables 2 and 3

**Fig. 14:**
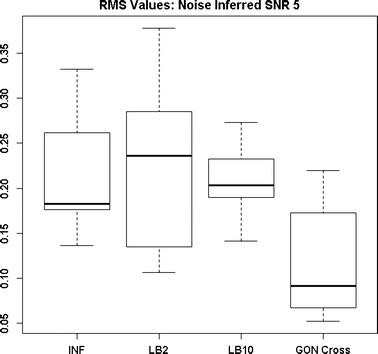
Distribution of RMS values in function space, calculated on the residuals of the true signal (signal produced with true parameters and no observational error) minus the signal produced with the estimate of the parameters, for the protein signalling transduction pathway (Eqs. 38–42). Within each section, the* boxplots* from* left to right* are: LB2 method, INF method, LB10 method, and GON method using cross validation for inferring the penalty parameter. The average signal to noise ratio for each “species” is 5. The standard deviation of the observational noise is inferred. For definitions, see Tables 2 and 3

Figures 13 and 14 show that the GON Cross method is slightly outperforming the INF, LB2 and LB10 methods, with RMS distributions that are on average 0.1 units lower.

The wider range of estimates of the parameters (as well as the long tails in the posterior distributions in Figs. 1, 2 and 3), for the INF, LB2 and LB10 methods, were observed when occasionally the time course signals would flatten. An inspection of Eq. (17) reveals that when $$f_{s}(\mathbf X ,\varvec{\theta },\mathbf t ) = \mathbf{0}$$$$\forall s$$, then $$p(\mathbf X |\varvec{\theta },\varvec{\phi },\varvec{\eta },\varvec{\gamma })$$ is maximised at $$\mathbf x _{s} = {\varvec{\phi }_s}$$$$\forall s$$. This corresponds to a flattening of the true concentration profiles, which usually can be assumed to be a poor fit to the data. Hence, this flattening should be discouraged by the likelihood term $$p(\mathbf Y |\mathbf X ,\varvec{\sigma })$$ in Eq. (4). However, for $$\varvec{\sigma } \gg \varvec{\sigma }_{\mathrm {True}}$$ (where $$\varvec{\sigma }_{\text {True}}$$ is the unknown true standard deviation of the observational error of the signals), the likelihood term is effectively switched off, which will allow the system to converge to a high probability attractor state corresponding to $$\mathbf x _{s} = {\varvec{\phi }_s}$$. In practice, we observe this effect for $$\varvec{\sigma }$$ exceeding $$\varvec{\sigma }_{\text {True}}$$. This attractor state is further self-enforcing by driving the length scales included in the GP hyperparameters $$\varvec{\eta }$$ to very large values, as we have observed in our simulations. Obviously, $$\mathbf x _{s} = {\varvec{\phi }_s}$$ is unrealistic. To test whether holding the standard deviation of the noise at the true value prevents the Markov chains from being driven to this unrealistic attractor state, we repeated the simulations of the comparison to GON and GON Cross, for the Fitz–Hugh Nagumo system and protein signalling transduction pathway for signal to noise ratios of 10 and 5. We held the standard deviation of the noise at the value that was used to generate the data, where in practice this could be estimated through a standard GP regression. We used the true value in order to observe whether this approach affects the results and to what extent, under the most favourable conditions.

**Fig. 15:**
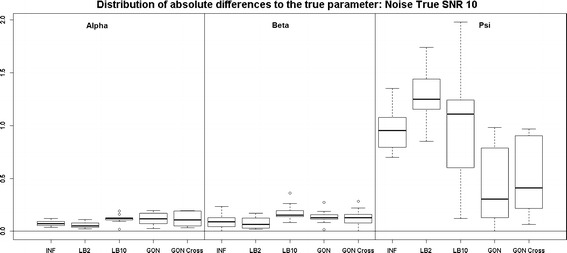
*Boxplots* of the distributions of the absolute differences of an estimate to the true parameter over 10 datasets. The three sections from* left to right* represent the parameters $\alpha$, $\beta$ and $\psi$ from the Fitz–Hugh Nagumo model (Eqs. 36, 37). Within each section, the* boxplots* from* left to right* are: LB2 method, INF method, LB10 method, GON method and GON method using cross validation for inferring the penalty parameter. The average signal to noise ratio for each “species” is 10. The standard deviation of the observational noise is held at the true value. For definitions, see Tables 2 and 3

**Fig. 16:**
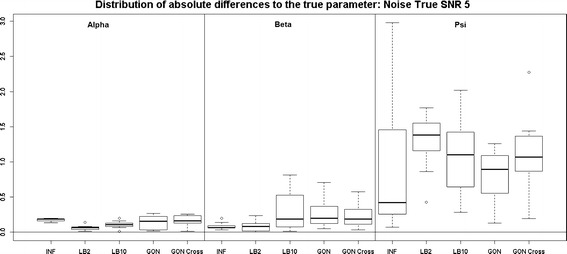
*Boxplots* of the distributions of the absolute differences of an estimate to the true parameter over 10 datasets. The three sections from* left to right* represent the parameters $\alpha$, $\beta$ and $\psi$ from the Fitz–Hugh Nagumo model (Eqs. 36, 37). Within each section, the* boxplots* from* left to right* are: LB2 method, INF method, LB10 method, GON method and GON method using cross validation for inferring the penalty parameter. The average signal to noise ratio for each “species” is 5. The standard deviation of the observational noise is held at the true value. For definitions, see Tables 2 and 3

Examining Fig. 15, where now the standard deviation of the noise is held fixed at the true value, the INF, LB2, LB10, GON and GON Cross methods perform similarly for the first 2 parameters and the GON and GON Cross do about 1 unit of absolute diferrence to the true parameter better for the 3rd. When the noise is increased, Fig. 16, the methods produce estimates that are similar to one another for all three parameters.

**Fig. 17:**
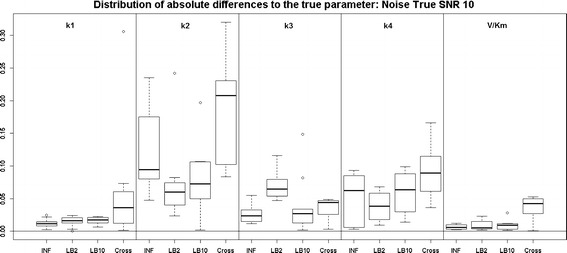
*Boxplots* of the distributions of the absolute differences of an estimate to the true parameter over 10 datasets. The 5 sections from* left to right* represent the parameters for the protein signalling transduction pathway, Eqs. (38–42). Within each section, the* boxplots* from left to right are: LB2 method, INF method, LB10 method and GON method using cross validation for inferring the penalty parameter. The average signal to noise ratio for each “species” is 10. The standard deviation of the observational noise is held at the true value. For definitions, see Tables 2 and 3

**Fig. 18:**
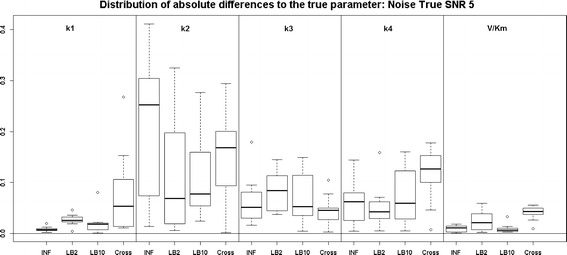
*Boxplots* of the distributions of the absolute differences of an estimate to the true parameter over 10 datasets. The 5 sections from* left to right* represent the parameters for the protein signalling transduction pathway, Eqs. (38–42). Within each section, the* boxplots* from* left to right* are: LB2 method, INF method, LB10 method and GON method using cross validation for inferring the penalty parameter. The average signal to noise ratio for each “species” is 5. The standard deviation of the observational noise is held at the true value. For definitions, see Tables 2 and 3

For the protein signalling transduction pathway, Eqs. (38–42), Fig. 17 shows that the INF, LB2 and LB10 methods perform on average 0.075 units in absolute value to the true parameter better than GON Cross, over the different parameters. Similarly, in Fig. 18, INF, LB2 and LB10 perform roughly 0.07 units better in absolute distance to the true parameter than GON Cross.

**Fig. 19:**
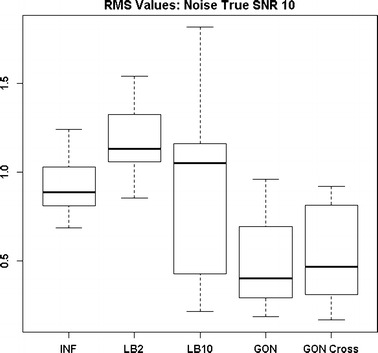
Distribution of RMS values in function space, calculated on the residuals of the true signal (signal produced with true parameters and no observational error) minus the signal produced with the estimate of the parameters, for the Fitz–Hugh Nagumo model (Eqs. 36, 37). Within each section, the* boxplots* from* left to right* are: LB2 method, INF method, LB10 method, GON method and GON method using cross validation for inferring the penalty parameter. The average signal to noise ratio for each “species” is 10. The standard deviation of the observational noise is held at the true value. For definitions, see Tables 2 and 3

**Fig. 20:**
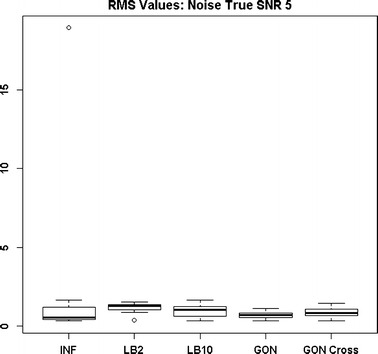
Distribution of RMS values in function space, calculated on the residuals of the true signal (signal produced with true parameters and no observational error) minus the signal produced with the estimate of the parameters, for the Fitz–Hugh Nagumo model (Eqs. 36, 37). Within each section, the* boxplots* from* left to right* are: LB2 method, INF method, LB10 method, GON method and GON method using cross validation for inferring the penalty parameter. The average signal to noise ratio for each “species” is 5. The standard deviation of the observational noise is held at the true value. For definitions, see Tables 2 and 3

The RMS distributions in Fig. 19 show that the GON and GON Cross methods are producing slightly better estimates, reflected by the distributions being around 0.5 units in RMS lower. For the increased noise scenario, Fig. 20, all methods are performing similarly.

**Fig. 21:**
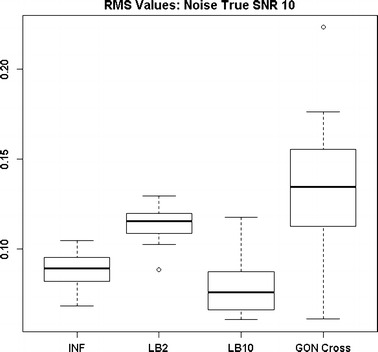
Distribution of RMS values in function space, calculated on the residuals of the true signal (signal produced with true parameters and no observational error) minus the signal produced with the estimate of the parameters, for the protein signalling transduction pathway (Eqs. 38–42). Within each section, the* boxplots* from* left to right* are: LB2 method, INF method, LB10 method and GON method using cross validation for inferring the penalty parameter. The average signal to noise ratio for each “species” is 10. The standard deviation of the observational noise is held at the true value. For definitions, see Tables 2 and 3

**Fig. 22:**
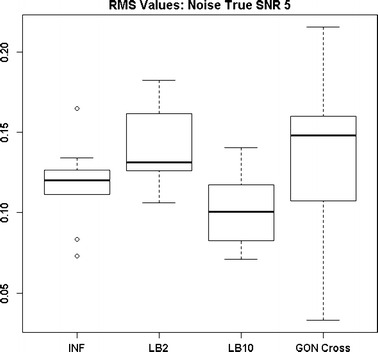
Distribution of RMS values in function space, calculated on the residuals of the true signal (signal produced with true parameters and no observational error) minus the signal produced with the estimate of the parameters, for the protein signalling transduction pathway (Eqs. 38–42). Within each section, the* boxplots* from* left to right *are: LB2 method, INF method, LB10 method and GON method using cross validation for inferring the penalty parameter. The average signal to noise ratio for each “species” is 5. The standard deviation of the observational noise is held at the true value. For definitions, see Tables 2 and 3

In Fig. 21, we can see that the INF, LB2 and LB10 methods outperform the GON Cross method, shown by smaller RMS distributions that are roughly 0.05 units smaller. In Fig. 22, the INF and LB10 methods do better than LB2 and GON Cross with RMS values on average 0.05 units smaller.

## Discussion

We have modified a recently developed gradient matching approach for systems biology (INF) by combining it with a parallel tempering scheme for the gradient mismatch parameter (C&S). We have also carried out a comparative evaluation of this new method with various state-of-the-art gradient matching methods. These methods are based on different inference approaches and statistical models, namely: non-parametric Bayesian statistics using GPs (INF, LB2, LB10), splines-based smooth functional tempering (C&S), hierarchical regularisation using splines interpolation (RAM), and penalised likelihood based on reproducing kernel Hilbert spaces (GON). Our set-ups have also allowed us to compare the opposing paradigms of Bayesian inference (INF) versus parallel tempering (LB2, LB10) of the slack parameters controlling the amount of mismatch between the gradients.

In one case, when the number of observations was very high (higher than what would be expected in these types of experiments) and the tuning parameters were finely adjusted (which is time-consuming in practice), the C&S method does well. When the dataset size was reduced, all settings for this method deteriorated significantly, including the previous tuning setting that performed well. It is also important to note that the particular settings that we found to be optimal were different than in the original paper, which highlights the sensitivity and lack of robustness in the splines based method.

The GON and GON Cross methods produce estimates that are close to the true parameters in terms of absolute uncertainty. For the Fitz-Hugh Nagumo ODE model, the method outperforms the other schemes for one parameter, in the case when the signal to noise ratio was 10 and 25 datapoints were generated. For the protein signalling transduction pathway, however, this method is outperformed by INF, LB2 and LB10. This method also has a drawback to practical implementation, on non-simulated data. The method, which uses a classical approach to parameter estimation (producing point estimates), cannot immediately produce confidence intervals for the parameters and so quantifying the uncertainty in the parameter estimates will be more difficult. For simulated data, this is not an issue, since it is possible to generate multiple datasets and quantify the accuracy of the method by observing the results across all datasets. In practice however, this is not available. One would need to rely on other processes, such as bootstrapping, and the effect on the accuracy and computational time is something that needs to be investigated.

The INF method performs reasonably by producing results close to the true parameters across the scenarios that we have examined. However, this method’s decrease in uncertainty is at the expense of bias.

The LB2 and LB10 methods show good performance across the set-ups. The parameter inference is accurate across the different ODE models and the different settings of those models. The parallel tempering schedule has proven to be quite robust, as the methods perform similarly across the various set-ups.

For some simulations, we noticed a flattening of the time course signals for INF, LB2 and LB10. The uncertainty in the signals reduced the accuracy in the methods. In order to achieve a robust method that provides accurate parameter estimation, we examined holding the standard deviation at the true value. In this case, the GON and GON Cross outperformed INF, LB2 and LB10 on one parameter in the Fitz-Hugh Nagumo system, when the signal to noise ratio was 10. For the signal to noise ratio setting of 5, the methods all performed similarly. The INF, LB2 and LB10 methods outperform the GON Cross method for the protein signalling transduction pathway. Holding the standard deviation of the noise at the true value, for the INF, LB2 and LB10 methods, stops the likelihood term from effectively being switched off and prevents the flattening. In practice, this parameter could be estimated by a standard GP regression, in order to fix the standard deviation of the noise when the true value is unknown. This is a somewhat heuristic fix to the problem however, and a general robust solution should be the focus for future research.

It is also important to note that the methods in this paper were not derived in order to operate with a particular ODE model. The results therefore, should be similar across ODE type. We have seen evidence of this in other ODE systems, like the Lorenz attractor and the Lotka-Volterra predator-prey model, which are less relevant to the field of biomedical engineering, though, and are thus beyond the scope of the present paper.

## Conclusions

The combination of adaptive gradient matching using GPs from Dondelinger et al. [4] and a parallel tempering scheme for the gradient mismatch parameter from Campbell and Steele [5], has yielded a method that provides accurate parameter estimates for ODEs when the true standard deviation of the noise is known. This method performs well across ODE models and variation of the scheduling of the tempered mismatch parameter.

We have found that the method in Dondelinger et al. [4] provides accurate estimation, although the decrease in uncertainty is at the expense of bias. The method in Campbell and Steele [5] shows a lack of robustness, due to the difficulty in configuring the splines settings. For the method in Ramsay et al. [3], we found it was outperformed by the other methods we looked at. The method in González et al. [14] is accurate and robust, but can be outperformed by Dondelinger et al. [4] and the proposed method in this paper. For a signal to noise ratio of 10 on the Fitz-Hugh Nagumo system, the González et al. [14] method is able to outperform the method in Dondelinger et al. [4] and our new method, for one parameter. We found that using cross validation as opposed to AIC for the González et al. [14] method, to estimate the penalty parameter, yielded results that were more robust.

In order to avoid a potential drawback to our proposed method, we hold the standard deviation of the noise at the true value, to avoid the signals deviating from the data and flattening. This remedy was found to lead to a significant improvement over the method with a flexible standard deviation of the error. In practice, the standard deviation of the noise could be estimated, for example by a standard GP regression, and general approaches to this should be the focus of future research.

## Footnotes

1 A summary of the notation used throughout this paper can be found in Table 1.

## References

[1] Calderhead B, Girolami MA, Lawrence ND. Accelerating Bayesian inference over non-linear differential equations with Gaussian processes. Neural Inf Process Syst (NIPS). 2008;22.

[2] Liang H, Wu H (2008). Parameter estimation for differential equation models using a framework of measurement error in regression models. J Am Stat Assoc.

[3] Ramsay JO, Hooker G, Campbell D, Cao J (2007). Parameter estimation for differential equations: a generalized smoothing approach. J R Statist.

[4] Dondelinger F, Filippone M, Rogers S, Husmeier D. ODE parameter inference using adaptive gradient matching with Gaussian processes. The 16th Int Conf Artif Intell Stat (AISTATS) 31 JMLR. 2013:216–28.

[5] Campbell D, Steele RJ (2012). Smooth functional tempering for nonlinear differential equation models. Stat Comput.

[6] Adon NA, Jabbar MH, Mahmud F. FPGA implementation for cardiac excitation-conduction simulation based on FitzHugh-Nagumo model. 5th Int Conf Biomed Eng Vietnam. 2015;46.

[7] Duckett G, Barkley D (2000). Modeling the dynamics of cardiac action potentials. Phys Rev Lett.

[8] Goktepe S, Kuhl, E. Computational modeling of cardiac electrophysiology: a novel finite element approach. Int J Numer Methods Eng. 2009. 10.1002/cnm.2565 PMC4567385 23798328

[9] Bruggemeier B, Schusterreiter C, Pavlou H, Jenkins N, Lynch S, Bianchi A, Cai X. Improving the utility of drosophila melanogaster for neurodegenerative disease research by modelling courtship behaviour patterns. Report summarising the outcomes from the UK NC3R’s and POEM’s meeting. 2014.

[10] Vivekanandan S, Emmanuel DS, Kumari R. Propogation of action potential for Hansen’s disease affected nerve model using FitzHugh Nagumo like excitation. J Theor Appl Inf Technol. 2013.

[11] Martin GS. Cell signaling and cancer. Meeting review. Cancer. 2003. 10.1016/s1535-6108(03)00216-2 14522250

[12] Kim EK, Choi E-J (2010). Pathological roles of mapk signaling pathways in human diseases. Biochimica et Biophysica Acta (BBA)–molecular basis of disease.

[13] Macdonald B, Husmeier D (2015). Computational inference in systems biology. Bioinformatics and Biomedical Engineering:Third International Conference, IWBBIO. Proceedings, Part II. Series: Lecture Notes in Computer Science, vol. 9044.

[14] González J, Vujačić I, Wit E (2013). Inferring latent gene regulatory network kinetics. Stat Appl Genet Mol Biol.

[15] Macdonald B, Husmeier D (2015). Gradient matching methods for computational inference in mechanistic models for systems biology: a review and comparative analysis. Front Bioeng Biotechnol.

[16] Solak E, Murray-Smith R, Leithead WE, Leith DJ, Rasmussen CE. Derivative observations in Gaussian process models of dynamic systems. Adv Neural Inf Process Syst. 2003; 9–14.

[17] Holsclaw, T., Sansó B, Lee HKH, Heitmann K, Habi S, Higdon D, Alam U. Gaussian process modeling of derivative curves. Technometrics. 2011.

[18] Bishop CM. Pattern recognition and machine learning. Berlin: Springer; 2006.

[19] Murray I, Adams R. Slice sampling covariance hyperparameters of latent gaussian models. Adv Neural Inf Process Syst (NIPS); 2010:23.

[20] Friel N, Pettitt AN (2008). Marginal likelihood estimation via power posteriors. J R Stat Soc.

[21] Murphy KP. Machine learning. A probabilistic perspective. The MIT Press. 2012.

[22] Aronszajin N. Green’s functions and reproducing kernels. Proceedings of the Symposium on spectral theory and differential problems; 1951:355–411.

[23] Rasmussen CE, Williams CKI. Gaussian processes for machine learning. The MIT Press; 2006.

[24] FitzHugh R (1961). Impulses and physiological states in models of nerve membrane. Biophys J.

[25] Nagumo JS, Arimoto S, Yoshizawa S (1962). An active pulse transmission line simulating a nerve axon. Proc Inst Radio Eng.

[26] Vyshemirsky V, Girolami MA (2008). Bayesian ranking of biochemical system models. Bioinformatics.

